# Non-antipsychotic medicines and modified electroconvulsive therapy are risk factors for hospital-acquired pneumonia in schizophrenia patients

**DOI:** 10.3389/fpsyt.2022.1071079

**Published:** 2023-01-13

**Authors:** Yan Yang, Di Kong, Qiwen Li, Wei Chen, Guocheng Zhao, Xi Tan, Xincheng Huang, Zipeng Zhang, Can Feng, Min Xu, Ying Wan, Mi Yang

**Affiliations:** ^1^The Fourth People's Hospital of Chengdu, Chengdu, Sichuan, China; ^2^MOE Key Lab for Neuroinformation, The Clinical Hospital of Chengdu Brain Science Institute, University of Electronic Science and Technology of China, Chengdu, China; ^3^School of Life Science and Technology, University of Electronic Science and Technology of China, Chengdu, China

**Keywords:** schizophrenia, hospital-acquired pneumonia, modified electroconvulsive therapy, benzodiazepines, mood stabilizers

## Abstract

**Background:**

Hospital-acquired pneumonia (HAP) has a significant and detrimental impact on schizophrenia patients. Non-antipsychotic medicines and modified electroconvulsive therapy (MECT) are frequently used in conjunction with antipsychotics to treat schizophrenia. Whether non-antipsychotic medicines or MECT are risk factors for HAP in schizophrenia treated with antipsychotics is still unknown.

**Methods:**

Patients with schizophrenia who were admitted to the Fourth People's Hospital of Chengdu between January 2015 and April 2022 were included in this retrospective cohort study. Individuals with HAP were 1:1 matched to individuals without HAP (non-HAP) using propensity score matching (PSM). The risk factors for HAP were analyzed by comparing the two groups.

**Results:**

A total of 7,085 schizophrenia patients were included in this study, with a mean age of 39.77 ± 14.45 years. 193 patients developed HAP on an average of 22.26 ± 21.68 days after admission with an incidence of 2.73%. After 1:1 PSM, 192 patients from each group (HAP and non-HAP) were included. The HAP group had significantly more patients with MECT and taking benzodiazepines, antidepressants, mood stabilizers, and anti-parkinsonians both before and after PSM by *Bonferroni* correction (*P* < 0.001). Multivariate logistic regression analysis showed that, combined with antipsychotics, non-antipsychotic medicines including benzodiazepines (OR = 3.13, 95%CI = 1.95-5.03, *P* < 0.001), mood stabilizers (OR =3.33, 95%CI =1.79–6.20, *P* < 0.001) and MECT (OR =2.58, 95%CI =1.49–4.46, *P* = 0.001) were associated with a significantly increased incidence of HAP.

**Conclusion:**

The incidence of HAP in schizophrenia patients in our cohort was 2.73%. MECT and non-antipsychotic medicines, including benzodiazepines and mood stabilizers were risk factors for HAP in schizophrenia patients treated with antipsychotics.

## Introduction

Schizophrenia is a severe mental disorder with various symptom domains, including positive, negative, and cognitive symptoms, and it remains one of the most challenging disorders to treat (1). Antipsychotic medication is the primary pharmacological treatment used, but clinicians often find it necessary to use it in conjunction with non-antipsychotic medicines or modified electroconvulsive therapy (MECT) (1–3). Several non-antipsychotic medicines are commonly used. Antidepressants are used to improve negative symptoms, and mood stabilizers are used to incapacitate mood instability. In addition, benzodiazepines are used for comorbid anxiety or distress, and anti-Parkinsonians are used in patients with parkinsonism as a side effect of antipsychotic therapy (4–8). In addition, MECT is currently considered an effective treatment option to treat schizophrenia, especially in patients with drug resistance, aggression, catatonia, severe depression, or suicidal behavior (9).

During the COVID-19 pandemic, pneumonia in schizophrenia patients has received increased attention (10, 11). People with schizophrenia are prone to develop hospital-acquired pneumonia (HAP), which refers to new pneumonia occurring more than 48 h after admission in nonincubated patients (12–14). Han et al. (13) found that the incidence of HAP was 1.80% in patients with schizophrenia-spectrum disorder. Moreover, Yang et al. (14) reported that 7.8% of middle-aged and elderly schizophrenia had HAP. HAP can increase the cost of treatment, lengthen the duration of hospital stays, and cause a significant increase in morbidity and mortality (12). Some risk factors for HAP have been reported, such as aging, obesity, alcoholism, smoking, underlying diseases, aspiration, malnutrition, being bedridden for a long time, and the medical environment (15). In recent studies, the use of first-generation antipsychotics or second-generation antipsychotics has been identified as a risk factor for HAP (14, 16). However, the correlation between non-antipsychotic medicines or MECT and HAP in patients with schizophrenia under antipsychotic treatment remains unclear.

A recent review has reported that psychotropic drugs, including antipsychotic and non-antipsychotic drugs, are associated with an increased risk of pneumonia in the elderly (17). MECT is a medical procedure that induces seizures to treat mental disease and the main adverse reactions are cognitive dysfunction, headache, nausea, vomiting, mild anxiety, and fever. Some of these adverse reactions may increase the incidence of pneumonia (18). Furthermore, in our past clinical observation, we found that some patients with schizophrenia might be susceptible to HAP after MECT treatment in our hospital. Although both non-antipsychotics and MECT are common treatment strategies, few studies have examined the impact of these factors on HAP occurrence. Therefore, in this study, we carried out a retrospective cohort study to investigate whether non-antipsychotic medicines or MECT are risk factors for HAP in schizophrenia patients who are routinely receiving antipsychotics.

## Materials and methods

### Study design and ethics statement

This retrospective cohort study was performed at the Fourth People's Hospital of Chengdu, a large-scale psychiatric hospital in southwest China. Patients with schizophrenia aged 14–75 years were enrolled in the study from January 2015 to April 2022, and all schizophrenics were in closed wards. The diagnosis of schizophrenia was consistent with ICD-10. The exclusion criterion included patients with infectious diseases at admission, a short stay (<3 days) in the hospital, and incomplete laboratory results and treatments. Included patients were divided into the HAP group and non-HAP group according to whether they had developed HAP. The diagnosis of HAP required all of the following conditions: fever, respiratory decline, new lung infiltrates on chest imaging, and a productive cough (12). This study was designed retrospectively. Written informed consent was waived, and the study was approved by the Institutional Review Committee of the Fourth People's Hospital of Chengdu.

### Data collection and propensity score matching (PSM)

All data were collected from the electronic medical record information system of our hospital. The following data were collected: age, sex, diabetes status, hypertension status, length of hospital stay, routine blood tests and serum biochemical examinations at admission, medications, and whether they had MECT or HAP. After data extraction, PSM analysis was performed with a caliper of 0.02 using State software (version 15.0, State Corporation, College Station, TX) by a 1:1 nearest neighbor matching method. The covariates included sex, age, diabetes, hypertension, direct bilirubin, indirect bilirubin, total bilirubin, uric acid, total cholesterol, high-density lipoprotein, low-density lipoprotein, triglycerides, red blood cell count, hemoglobin, white blood cell count, monocyte count, lymphocyte count, basophil count, eosinophil count, and platelet count.

### Statistical analysis

In this study, continuous variables were summarized as means ± standard deviations, and categorical variables were summarized as frequencies or percentages (%). To compare the differences between the HAP and the non-HAP groups, the Student's *t*-test and chi-square test were performed both before and after PSM. A chi-square test was performed to analyze the differences in medication use and MECT between the HAP and non-HAP groups. The *Bonferroni* method was used to adjust the level of α for pairwise comparison (α = 0.05/26 = 0.0019). Logistic regression analysis was used to examine the impact of medications and MECT on HAP incidence, and an odds ratio (OR) was calculated with a 95% confidence interval (CI). Statistical analysis was performed using SPSS version 25.0 for Windows (SPSS Inc, Chicago, IL, USA), and P <0.05 was used as the cutoff for statistical significance.

## Results

### Baseline characteristics

**Figure 1:**
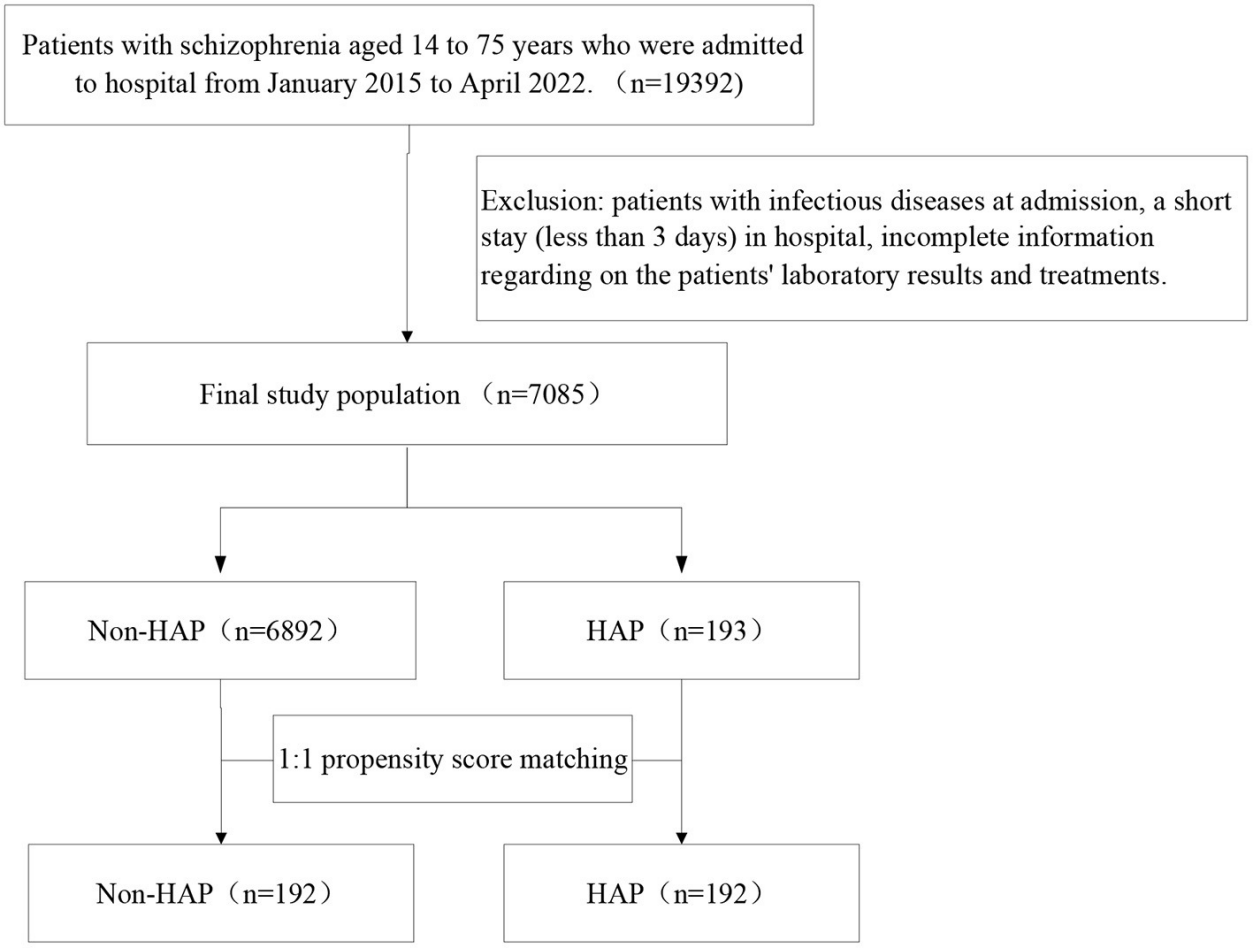
The study design flow chart.

A total of 19,392 inpatients with schizophrenia were screened and 7,085 participants were included (Figure 1). Among all patients, the median age was 39.77 ± 14.45 years and 3,119 (45.2%) were male. All patients were treated with antipsychotics. The patients were then divided into the HAP group (*n* = 193) and the non-HAP group (*n* = 6,892), and the incidence of HAP was 2.73%. The median time to HAP occurrence was 22.26±21.68 days after admission.

### Characteristics of HAP group and non-HAP group before and after PSM

Personal characteristics of the HAP group and non-HAP group before and after PSM are summarized in Table 1. Before PSM, the gender breakdowns of the HAP group and the non-HAP group were different (χ^2^=11.24, *P* = 0.001), and the age of the HAP group was higher than that of the non-HAP group (*t* = −3.32, *P* = 0.001). More people in the HAP group had diabetes (χ^2^=70.00, P<0.001) and hypertension (χ^2^= 56.16, *P* < 0.001). There were also statistically significant differences between the two groups in lab results, including blood direct bilirubin, total cholesterol, monocyte count, lymphocyte count, white blood cell count, basophil count, eosinophil count, red blood cell count, and platelet count at admission (*P* < 0.05) (Table 1). Monocyte count and lymphocyte count were also statistically significant differences between the two groups after *Bonferroni* correction (*P* < 0.001). After 1:1 PSM, the matched HAP (*n* = 192) and non-HAP groups (n = 192) were generated. The baseline characteristics were well-balanced, and there were no statistically significant differences between the two groups in age, sex, diabetes, hypertension, routine blood tests, or serum biochemical examinations at admission.

**Table 1:** Personal characteristics of patients with schizophrenia with and without HAP (before and after PSM).

	**Original cohort (before PSM**, ***n*** = **7,085)**				**Matched cohort (after PSM**, ***n*** = **384)**			
	**Non-HAP (*****n** =* **6,892)**	**HAP (*****n** =* **193)**	***t/** χ^2^*	***P***	**Non-HAP (*****n** =* **192)**	**HAP (*****n** =* **192)**	***t/** χ^2^*	***P***
**Gender** ^*^								
Female	3,803 (55.18%)	83 (43.01%)	11.24	0.001	82 (42.71%)	83 (43.23%)	0.01	0.918
Male	3,089 (44.82%)	110 (56.99%)			110 (57.29%)	109 (56.77%)		
**Age** ^*^	39.65 ± 14.35	43.86 ± 17.43	−3.32	0.001	43.32 ± 14.85	43.94 ± 17.43	−0.38	0.706
14–17	206 (2.99%)	7 (3.63%)	37.83	0.000	5 (2.60%)	7 (3.65%)	4.94	0.176
18–44	3,986 (57.84%)	85 (44.04%)			87 (45.31%)	84 (43.75%)		
45–59	2,004 (29.08%)	56 (29.02%)			70 (36.46%)	56 (29.17%)		
60–75	696 (10.10%)	45 (23.32%)			30 (15.63%)	45 (23.44%)		
**Diabetes** ^*^								
Yes	209 (3.03%)	27 (13.99%)	70.00	0.000	23 (11.98%)	27 (14.06%)	0.37	0.544
No	6,683 (96.97%)	166 (86.01%)			169 (88.02%)	165 (85.94%)		
**Hypertension** ^*^								
Yes	185 (2.68%)	23 (11.92%)	56.16	0.000	25 (13.02%)	23 (11.98%)	0.10	0.758
No	6,707 (97.32%)	170 (88.08%)			167 (86.98%)	169 (88.02%)		
DB (μmol/L)	3.31 ± 2.54	3.81 ± 2.51	−2.69	0.008	3.61 ± 2.47	3.78 ± 2.49	−0.67	0.501
IB (μmol/L)	10.49 ± 5.98	10.67 ± 6.12	−0.43	0.666	11.00 ± 7.10	10.57 ± 5.95	0.65	0.518
TB (μmol/L)	13.92 ± 7.75	14.36 ± 7.91	−0.75	0.454	14.41 ± 9.37	14.22 ± 7.70	0.22	0.824
TC (mmol/L)	4.57 ± 1.06	4.39 ± 1.06	2.39	0.017	4.45 ± 1.05	4.41 ± 1.02	0.37	0.713
TG (mmol/L)	1.26 ± 1.04	1.19 ± 0.71	0.94	0.347	1.20 ± 0.70	1.19 ± 0.72	0.17	0.866
HDL (mmol/L)	1.50 ± 0.37	1.49 ± 0.39	0.54	0.590	1.50 ± 0.39	1.49 ± 0.39	0.42	0.673
LDL (mmol/L)	2.28 ± 0.69	2.27 ± 0.67	0.23	0.821	2.28 ± 0.69	2.67 ± 0.67	0.18	0.857
Albumin (g/L)	41.72 ± 3.77	41.34 ± 4.37	1.20	0.232	41.57 ± 4.40	41.31 ± 4.37	0.57	0.566
UA (μmol/L)	347.42 ± 114.55	352.00 ± 123.31	−0.55	0.585	360.80 ± 126.38	351.48 ± 123.43	0.73	0.465
WBC (10^9^/L)	7.10 ± 2.40	7.60 ± 2.81	−2.45	0.015	8.02 ± 3.05	7.61 ± 2.81	1.38	0.169
MON (10^9^/L)^*^	0.43 ± 0.17	0.48 ± 0.21	−3.47	0.001	0.51 ± 0.24	0.48 ± 0.21	1.22	0.223
LYM (10^9^/L)^*^	1.85 ± 0.67	1.67 ± 0.65	3.56	0.000	1.68 ± 0.65	1.67 ± 0.65	0.11	0.911
BAS (10^9^/L)	0.03 ± 0.01	0.03 ± 0.02	1.70	0.091	0.030 ± 0.02	0.03 ± 0.02	1.39	0.166
EOS (10^9^/L)	0.12 ± 0.13	0.10 ± 0.14	1.89	0.058	0.11 ± 0.15	0.10 ± 0.14	0.72	0.474
RBC (10^12^/L)	4.53 ± 0.56	4.49 ± 0.59	1.17	0.243	4.50 ± 0.59	4.49 ± 0.59	0.18	0.857
PLT (10^9^/L)	214.24 ± 65.20	201.87 ± 74.89	2.27	0.024	201.72 ± 69.54	202.36 ± 74.78	−0.09	0.931
HGB (g/L)	135.09 ± 17.26	135.86 ± 19.41	−0.61	0.540	136.01 ± 18.18	135.84 ± 19.50	0.09	0.931

DB, Direct Bilirubin; IB, Indirect Bilirubin; TB, Total Bilirubin; TC, Total Cholesterol; TG, Triglycerides; HDL; High-density lipoprotein; LDL, Low-density Lipoprotein; UA, Uric Acid; WBC, white blood cell count; MON, monocyte count; LYM, lymphocyte count; BAS, basophil count; EOS, eosinophil count; RBC, red blood cell count; PLT, platelet count; HGB, hemoglobin.

^*^Indicate that the difference between groups was statistically significant after Bonferroni correction.

### Effect of non-antipsychotic medicines on HAP infection

After Bonferroni correction, The HAP group had significantly more patients taking benzodiazepines, antidepressants, mood stabilizers, and anti-parkinsonians both before and after PSM (*P* < 0.001) (Table 2). The multivariate logistic regression analysis revealed that except for antidepressants (OR = 3.01, 95%CI = 1.22–7.44, P = 0.017) and antiparkinsonians (OR =1.97, 95%CI = 1.22–3.17, *P* = 0.005), non-antipsychotic medicines including benzodiazepines (OR = 3.13, 95%CI = 1.95–5.03, *P* < 0.001), mood stabilizers (OR = 3.33, 95%CI = 1.79–6.20, *P* < 0.001) were significantly associated with an increased incidence of HAP in patients with schizophrenia treated with antipsychotics (*P* < 0.001) (Table 3).

**Table 2:** Association of non-antipsychotic medicines and MECT with HAP in patients with schizophrenia treated with antipsychotics.

		**Original cohort (before PSM**, ***n*** = **7,085)**						**Matched cohort (after PSM**, ***n*** = **384)**					
		**Non-HAP (*****N** =* **6892)**		**HAP(*****N** =* **193)**		*χ^2^*	***P***	**Non-HAP (*****N** =* **192)**		**HAP (*****N** =* **192)**		*χ^2^*	***P***
MECT	Yes	889	12.90%	62	32.12%	59.71	0.000	29	15.10%	61	31.77%	14.86	0.000
	No	6,003	87.10%	131	67.88%			163	84.90%	131	68.23%		
Benzodiazepines	Yes	3,124	45.33%	149	77.20%	76.74	0.000	88	45.83%	148	77.08%	39.58	0.000
	No	3,768	54.67%	44	22.80%			104	54.17%	44	22.92%		
Antidepressants	Yes	422	6.12%	26	13.47%	17.11	0.000	7	3.65%	26	13.54%	11.97	0.000
	No	6,470	93.88%	167	86.53%			185	96.35%	166	86.46%		
Mood stabilizers	Yes	777	11.27%	52	26.94%	44.62	0.000	18	9.38%	52	27.08%	20.20	0.000
	No	6,115	88.72%	141	73.06%			174	90.62%	140	72.92%		
Antiparkinsonians	Yes	1,903	27.61%	94	48.70%	41.27	0.000	48	25.00%	94	48.96%	23.65	0.000
	No	4,989	72.39%	99	51.30%			144	75.00%	98	51.04%		

**Table 3:** Multivariate logistic regression analysis of risk factors for HAP in patients with schizophrenia treated with antipsychotics.

	**B**	**SE**	**Wald chi-square**	***P***	**Exp(B)**	**95%CI**
MECT^*^	0.95	0.28	11.37	0.001	2.58	1.49–4.46
Benzodiazepines^*^	1.14	0.24	22.16	0.000	3.13	1.95–5.03
Antidepressants	1.10	0.46	5.72	0.017	3.01	1.22–7.44
Mood stabilizers^*^	1.20	0.32	14.40	0.000	3.33	1.79–6.20
Antiparkinsonians	0.68	0.24	7.80	0.005	1.97	1.22–3.17
Constant	−1.47	0.22	44.40	0.000	0.23	

^*^Indicate that the difference was statistically significant after the Bonferroni correction.

### Effect of MECT on HAP infection

Before PSM, of the 193 patients with HAP, 62 patients (32.12%) were treated with MECT. The HAP group had significantly more patients who underwent MECT than the non-HAP group after PSM (χ^2^= 14.86, *P* < 0.001). From the multivariate logistic regression analysis, receiving MECT was significantly correlated with HAP (OR = 2.58, 95%CI = 1.49–4.46, *P* = 0.001).

## Discussion

Antipsychotic monotherapy is a recommended treatment for schizophrenia by the American Psychiatric Association Practice Guideline, but the combination of antipsychotics with non-antipsychotics or MECT is common in practice (19–22). Studies that have focused on pneumonia or HAP in patients with schizophrenia have only focused on the effects of antipsychotics (16, 23–25). However, the combination of other psychiatric drugs or treatment methods also needs to be considered. To our knowledge, this is the first study to examine the incidence of HAP in patients with schizophrenia in a specialized psychiatric hospital and to investigate whether non-antipsychotic medicines or MECT are risk factors for HAP in patients with schizophrenia treated with antipsychotics. This retrospective cohort study found a 2.73% of HAP for hospitalized patients with schizophrenia, which was higher than the overall incidence of 1.6% for nonventilator hospital-acquired pneumonia in the United States (26). After *the Bonferroni* correction, we found the HAP group had significantly more patients with MECT and taking benzodiazepines, antidepressants, mood stabilizers, and anti-parkinsonians both before and after PSM (*P* < 0.001). Multivariate logistic regression analysis showed that MECT and non-antipsychotic medicines only benzodiazepines and mood stabilizers were independent risk factors for HAP in patients with schizophrenia treated with antipsychotics.

### Evidence and risk factors for HAP in schizophrenia treated with antipsychotics

It has been reported that benzodiazepines are effective drugs for anxiety, agitation, and insomnia (27). An antipsychotic-benzodiazepine combination treatment regimen is often necessary to control exacerbations of symptoms in the acute phase or severe psychotic relapses in patients with schizophrenia (5). Dublin et al. (28) and Iqbal et al. (29) suggested that benzodiazepines were not correlated with an increased risk of pneumonia. However, studies in recent years have had different results. Cheng et al. (30) reported a dose-dependent relationship between benzodiazepines and pneumonia in patients with schizophrenia and Taipale et al. (31) found that the use of benzodiazepine by patients with Alzheimer's disease was associated with an increased risk of pneumonia. A recent systematic review also reported that benzodiazepines and benzodiazepine-related drugs were associated with an increased pneumonia risk (32). Similar to recent reports, we found that benzodiazepine use increased the incidence of HAP in patients with schizophrenia treated with antipsychotics.

Adding a mood stabilizer is an option to reduce aggression or stabilize mood in patients with schizophrenia treated with antipsychotics (5). In this study, we found that the incidence of HAP was significantly higher in patients with schizophrenia who took mood stabilizers. Several studies have had similar results to ours. Taipale H et al. (33) found that mood stabilizers, including phenytoin, carbamazepine, valproic acid, and pregabalin, were associated with an increased risk of pneumonia. Han et al. (13) suggested that patients with mental disorders who received mood stabilizers had a higher likelihood of suffering HAP. Yang et al. (34) showed that patients treated with clozapine plus valproic acid (RR = 4.80, *P* < 0.001) and olanzapine plus carbamazepine (RR = 11.88, *P* < 0.01) had the highest risk of pneumonia.

Depressive symptoms are common in patients with schizophrenia, with an estimated incidence of about 25% (35). The addition of antidepressants to the treatment regimen of patients treated with antipsychotics is recommended to treat persistent symptoms, including depressive symptoms, negative symptoms, and other psychotic manifestations (5). Over the past decade, there have been conflicting reports regarding the relationship between the use of antidepressants and pneumonia. In 2007, Hennessy et al. (36) found that antidepressant use in elderly patients did not increase their risk of hospitalization for pneumonia or aspiration pneumonia. However, in 2018, Vozoris et al. (37) found that elderly patients using selective serotonin reuptake inhibitors/serotonin-noradrenaline reuptake inhibitors (SSRI/SNRI) had significantly higher rates of hospitalization for chronic obstructive pulmonary disease and pneumonia. In 2022, Kuo et al. (38) reported that the use of certain antidepressants of different classes was associated with pneumonia. We found that the HAP group had significantly more patients taking antidepressants, but multivariate logistic regression analysis showed no statistical difference. The differences between these findings as mentioned above may be a result of the different types or doses of antidepressants.

Among adults diagnosed with schizophrenia, 44% of anti-parkinsonian drugs were prescribed because of symptoms caused by antipsychotic treatment (20). Dies et al. (39) observed that patients treated with pramipexole had an increased rate of pneumonia (RR = 2.5,95%CI: 0.9–7.0) compared to placebo. In contrast, Ernst et al. (40) found that pramipexole did not increase the risk of pneumonia. We found that the HAP group had significantly more patients taking anti-parkinsonians, but multivariate logistic regression analysis showed no statistical difference. Since the use of anti-parkinsonians was related to the type and dose of antipsychotic drugs, more research is needed to investigate the interaction between antipsychotics and anti-parkinsonians on the occurrence of HAP.

Electroconvulsive therapy (ECT) has been used in psychiatry for more than 80 years (41). Traditional ECT involves passing an electrical current through the brain to cause a generalized seizure, but in modified ECT (MECT), anesthetics and muscle relaxants are applied before the treatment to alleviate side effects (42, 43). Although the therapeutic mechanism of MECT has not yet been fully elucidated, MECT is an effective and safe therapy for patients with schizophrenia if operating procedures are strictly followed. To our knowledge, this is the first report that MECT may increase the risk of HAP, with significantly higher rates of patients receiving MECT in the HAP group than in the non-HAP group after PSM (31.77 vs. 15.10%).

### Possible mechanisms for the association of medication and MECT with HAP

Drug-induced salivation, sedation, and anticholinergic effects may be responsible for HAP in patients with schizophrenia, which are the most common adverse effects of antipsychotics, antidepressants, benzodiazepines, mood stabilizers, and anti-parkinsonians (44). The mechanism may be mediated by several receptor mechanisms. Antipsychotics bind to dopaminergic, muscarinic 1 (M1), and histaminergic 1 (H1) receptors, resulting in sedation, anticholinergic effects, extrapyramidal symptoms, and hypersalivation. These symptoms may lead to dysphagia, aspiration, and pneumonia (45). In addition, antidepressants typically block the muscarinic (acetylcholine), histaminic (H1), dopaminergic (D2), alpha-1 adrenergic, and possibly serotonergic (5-HT2A) receptors, leading to salivation, sedation, and anticholinergic effects (46). Benzodiazepines act on the γ-aminobutyric acid (GABA_A_) receptors, and the most common adverse reaction is sedation, possibly leading to an increased risk of aspiration pneumonia (47). Mood stabilizers, especially antiepileptics, have been reported to have a sedative effect in elderly patients, which may increase the risk of aspiration and pneumonia (48, 49). Anti-parkinsonians such as trihexyphenidyl are commonly used in the treatment of Parkinsonian syndrome and for extrapyramidal reactions in schizophrenia, which have anticholinergic effects and can lead to malignant hyperthermia, both of which may increase the risk of HAP (48, 50).

The effects of MECT and medications on the immune system may also contribute to HAP in patients with schizophrenia. Serum cytokine concentration is closely associated with infections. High levels of interleukin (IL)-6 and IL-10 have been associated with poor prognosis in community-acquired pneumonia (51–53). A systematic review found that concentrations of tumor necrosis factor (TNF)-α, IL-1β, and IL-6 were elevated after a single session of MECT (54). Another meta-analysis revealed that MECT induced an increase in IL-6 levels and a potential decrease in TNF-α levels (55). Guloksuz et al. (56) suggested that repetitive MECT may downregulate immune activation, which may be associated with pneumonia. Psychotropic drugs have also been found to have both direct and indirect effects on the immune system that could lead to an increased risk of pneumonia (17, 57). Benzodiazepines have been reported to potentially increase pneumonia susceptibility as they have been found to activate certain GABA receptors on immune cells in mice (58).

It should be noted that catatonia is a typical condition of patients with schizophrenia who primarily required MECT and benzodiazepines treatment. Catatonia itself is a confounding factor in assessing whether MECT or benzodiazepines are risk factors, as they tend to induce aspiration pneumonia (59).

## Limitations

This study has several certain limitations. First, because this study is a retrospective study, no data were available such as body mass index, smoking, alcohol use, and hospital environment, which may be related to the risk of infection. Second, some potential confounding factors, such as physical complications, concomitant physical medications, and the severity of schizophrenia symptoms (e.g., catatonia) were missing from our analysis. Third, we did not analyze specific antipsychotics and dosages while studying the effects of MECT and non-antipsychotic medicines on HAP. Although exposure to both first-generation antipsychotics and second-generation antipsychotics is associated with an increased risk of pneumonia, clozapine is considered to be the most relevant. Therefore, the dose and type of antipsychotics should be taken into account in future research. Fourth, we provide evidence to support a causal relationship between MECT, non-antipsychotic medicines, and HAP, but the specific drug and mechanisms are still unclear. Finally, this is a single-center study, and a prospective multicenter study with a larger sample size is necessary to further validate our results.

## Conclusions

In this study, we found a HAP incidence of 2.73% in patients with schizophrenia. MECT and non-antipsychotic medicines including benzodiazepines and mood stabilizers were independent risk factors for HAP in patients with schizophrenia treated with antipsychotics.

## Data availability statement

The original contributions presented in the study are included in the article/supplementary material, further inquiries can be directed to the corresponding author.

## Ethics statement

The studies involving human participants were reviewed and approved by the Institutional Review Committee of the Fourth People's Hospital of Chengdu. Written informed consent for participation was not required for this study in accordance with the national legislation and the institutional requirements.

## Author contributions

YY and MY contributed to the conception and design of the study, performed the statistical analysis, and acquired the funding. DK, WC, XT, XH, and MX organized the database. YY wrote the first draft of the manuscript. QL, MY, ZZ, CF, and YW wrote sections of the manuscript. YY, GZ, and MY reviewed the manuscript. All authors contributed to the manuscript revision, read, and approved the submitted version.
